# Continuous erector spinae plane block versus thoracic epidural analgesia in video-assisted thoracic surgery: a study protocol for a prospective randomized open label non-inferiority trial

**DOI:** 10.1186/s13063-021-05275-9

**Published:** 2021-05-04

**Authors:** R. J. C. van den Broek, J. S. H. A. Koopman, J. M. C. Postema, N. J. Verberkmoes, K. J. Chin, R. A. Bouwman, B. J. B. Versyck

**Affiliations:** 1 Department of Anesthesiology and Pain Medicine, Catharina Hospital, Michelangelolaan 2, Eindhoven, 5623 EJ The Netherlands; 2 Department of Anesthesiology and Pain Medicine, Maasstad Hospital, Maasstadweg 21, Rotterdam, 3079 DZ the Netherlands; 3 Heart Center Catharina Hospital, Michelangelolaan 2, Eindhoven, 5623 EJ the Netherlands; 4 Department of Anesthesiology and Pain Medicine, Toronto Western Hospital, University of Toronto, 339 Bathurst St, Toronto, Ontario M5T 2S8 Canada; 5 Department of Anesthesiology and Pain Medicine, AZ Turnhout, Steenweg op Merksplas 44, 2300 Turnhout, Belgium

**Keywords:** Erector spinae plane block, Thoracic epidural analgesia, Regional anesthesia, Postoperative pain, Video-assisted thoracoscopic surgery, Pain management, Randomized controlled trial

## Abstract

**Background:**

Thoracic epidural analgesia is considered the gold standard for pain relief in video-assisted thoracoscopic surgery. This neuraxial technique blocks pain sensation by injecting a local anesthetic agent in the epidural space near the spinal cord to block spinal nerve roots. Recently, the erector spinae plane block has been introduced as a practical alternative to the thoracic epidural. This interfascial regional anesthesia technique interrupts pain sensation by injecting a local anesthetic agent in between the muscular layers of the thoracic wall. Several case series and three RCTs described it as an effective pain management technique in video-assisted thoracoscopic surgery (Scimia et al., Reg Anesth Pain Med 42:537, 2017; Adhikary et al., Indian J Anaesth 62:75–8, 2018; Kim, A randomized controlled trial comparing continuous erector spinae plane block with thoracic epidural analgesia for postoperative pain management in video-assisted thoracic surgery, n.d.; Yao et al., J Clin Anesth 63:109783, 2020; Ciftci et al., J Cardiothorac Vasc Anesth 34:444–9, 2020).

The objective of this study is to test the hypothesis that a continuous erector spinae plane block incorporated into an opioid-based systemic multimodal analgesia regimen is non-inferior in terms of the quality of postoperative recovery compared to continuous thoracic epidural local anesthetic-opioid analgesia in patients undergoing elective unilateral video-assisted thoracoscopic surgery.

**Methods:**

This is a prospective randomized open label non-inferiority trial. A total of 90 adult patients undergoing video-assisted thoracoscopic surgery will be randomized 1:1 to receive pain treatment with either (1) continuous erector spinae plane block plus intravenous patient-controlled analgesia with piritramide (study group) or (2) continuous thoracic epidural analgesia with a local anesthetic-opioid infusate (control group). All patients will receive additional systemic multimodal analgesia with paracetamol and non-steroidal anti-inflammatory drugs. The primary endpoint is the quality of recovery as measured by the Quality of Recovery-15 score. Secondary endpoints are postoperative pain as Numerical Rating Score scores, length of hospital stay, failure of analgesic technique, postoperative morphine-equivalent consumption, itching, nausea and vomiting, total operative time, complications related to surgery, perioperative hypotension, complications related to pain treatment, duration of bladder catheterization, and time of first assisted mobilization > 20 m and of mobilization to sitting in a chair.

**Discussion:**

This randomized controlled trial aims to confirm whether continuous erector spinae plane block plus patient-controlled opioid analgesia can equal the analgesic effect of a thoracic epidural local anesthetic-opioid infusion in patients undergoing video-assisted thoracoscopic surgery.

**Trial registration:**

Netherlands Trial Register NL6433. Registered on 1 March 2018. This trial was prospectively registered.

## Administrative information

The order of the items has been modified to group similar items (see http://www.equator-network.org/reporting-guidelines/spirit-2013-statement-defining-standard-protocol-items-for-clinical-trials/).

**Table:** 

Title {1}	Continuous erector spinae plane block versus thoracic epidural analgesia in video-assisted thoracic surgery: a study protocol for a prospective randomized open label non-inferiority trial
Trial registration {2a and 2b}.	Netherlands Trial Register NL6433 https://www.trialregister.nl/trial/6433
Protocol version {3}	Protocol version 5; 22/11/2019.
Funding {4}	This work was supported by the Stichting Onderzoeksfonds Catharina Ziekenhuis (Foundation Research Fund Catharina Hospital) (Project 2018-7). It will cover the cost for a research nurse who will enter the data in the database and publication costs.
Author details {5a}	R.J.C. (Renee) van den Broek MD (corresponding author) Department of Anesthesiology and Pain medicine, Catharina hospital Michelangelolaan 2, 5623 EJ Eindhoven, The Netherlands Renee.vd.broek@catharinaziekenhuis.nl J.S.H.A. (Seppe) Koopman MD, PhD, MSc Department of Anesthesiology and Pain Medicine, Maasstad Hospital Maasstadweg 21, 3079 DZ, Rotterdam, the Netherlands KoopmanJ@maasstadziekenhuis.nl J.M.C. (Jonne) Postema MD Department of Anesthesiology and Pain Medicine, Maasstad Hospital Maasstadweg 21, 3079 DZ, Rotterdam, the Netherlands PostemaJ@maasstadziekenuis.nl N.J. (Niels) Verberkmoes MD Heart Center Catharina Hospital Michelangelolaan 2, 5623 EJ Eindhoven, the Netherlands Niels.verberkmoes@catharinaziekenhuis.nl K.J. (Ki Jinn) Chin MBBS, FRCPC Department of Anesthesiology and Pain Medicine, Toronto Western Hospital, University of Toronto 339 Bathurst St, M5T 2S8 Toronto, Ontario, Canada gasgenie@gmail.com R.A. (Arthur) Bouwman MD, PhD Department of Anesthesiology and Pain Medicine, Catharina hospital Michelangelolaan 2, 5623 EJ Eindhoven, The Netherlands Arthur.bouwman@catharinaziekenhuis.nl B.J.B (Barbara) Versyck MD, PhD Department of anesthesiology and pain medicine, Catharina hospital Michelangelolaan 2, 5623 EJ Eindhoven, The Netherlands Department of Anesthesiology and Pain Medicine, AZ Turnhout Steenweg op Merksplas 44, 2300 Turnhout, België Barbara.versyck@azturnhout.be
Name and contact information for the trial sponsor {5b}	Catharina Hospital, board of directors Department research and education, Catharina hospital Michelangelolaan 2, 5623 EJ Eindhoven, The Netherlands +31 40 2396520
Role of sponsor {5c}	This funding source had no role in the design of this study and will not have any role during its execution, analyses, interpretation of the data, or decision to submit results The sponsor has no role in the design, execution, analysis, interpretation of the study or the decision to submit results. The role of the sponsor is according to GCP guidelines.

## Introduction

### Background and rationale {6a}

Thoracic epidural analgesia (TEA) is considered the gold standard analgesic technique for video-assisted thoracoscopic surgery (VATS) [1]. The invasiveness of this technique, the need for bladder catheterization due to transient impairment of bladder function, the rare but serious neurologic complications and the failure rates up to 30% [2] are the shortcomings of epidural analgesia. Also, neuraxial techniques are contra-indicated in patients using anticoagulation apart from acetylsalicylic acid and other non-steroidal anti-inflammatory drugs (NSAIDs) [3]. Sepsis and infection are relative contraindications [4]. This has fuelled the interest in alternatives to TEA. Alternatives include intercostal nerve blocks, paravertebral blocks, intrapleural catheters, local anesthetic infiltration, and systemic analgesia with one or more agents [1]. However, none of these techniques were able to replace the thoracic epidural as gold standard due to being “too technically challenging” [5] or providing “insufficient analgesia” [6].

The most recently described alternative to TEA is the erector spinae plane (ESP) block. The ESP block is a fascial plane block that aims to inject a local anesthetic agent within a plane beneath the erector spinae muscle [6]. The most significant advantages of the ESP block are its perceived simplicity and safety. The sonoanatomy is easily recognizable and there are no structures at risk of needle injury in the immediate vicinity [6]. The use of anticoagulation is not an absolute contra-indication to this technique [7, 8]. Multiple case reports have described its successful application for analgesia after VATS [9, 10], and two RCTs reported that a single-injection ESP block lowers pain scores after VATS compared to no intervention [11, 12]. Two other RCTs have found continuous ESP block to be non-inferior to paravertebral block and TEA for analgesia in VATS during the first 24 h postoperatively [13, 14]. However, further evidence from multicenter trials comparing continuous ESP block to TEA in VATS is required to confirm these preliminary findings.

### Objectives {7}

Our primary objective is to test the hypothesis that continuous ESP block incorporated into a multimodal analgesia regimen which includes intravenous patient-controlled opioid analgesia is non-inferior to continuous TEA with a local anesthetic-opioid infusate in patients undergoing elective unilateral VATS. Non-inferiority will be assessed in terms of the quality of postoperative recovery, as measured by the Quality of Recovery-15 (QoR-15) score on postoperative days (POD) 1 and 2 [15].

### Trial design {8}

This is an investigator-initiated prospective randomized open label non-inferiority trial. It is designed as a non-inferiority trial as the analgesic effectiveness of a successful TEA is unquestioned. Furthermore, it is not necessary to demonstrate the analgesic superiority of the ESP block because of the previously mentioned advantages it has over TEA in terms of feasibility and safety. A double-blinded study protocol is not possible as the differences between continuous ESP block and TEA will be readily apparent during the study period; hence, we have adopted an open-label study design.

## Methods: participants, interventions, and outcomes

### Study setting {9}

The study will be performed in the Catharina Hospital, Eindhoven, the Netherlands, and Maasstad Hospital, Rotterdam, the Netherlands. Both centers are large teaching hospitals.

### Eligibility criteria {10}

In order to be eligible to participate in this study, a subject must meet all of the following criteria: (1) age between 18 and 75 years old, (2) BMI between 18 and 30 kg/m^2^, (3) scheduled for elective VATS (complete VATS, as described by Swanson and Shigemura) [16, 17], and (4) written informed consent.

Exclusion criteria are as follows: (1) ASA status 4 or 5, (2) chronic opioid use, defined as > 3 months of opioid use (excluding tramadol and codeine), (3) renal or liver failure inhibiting the systematic use of paracetamol and/or NSAIDs, (4) contraindications to epidural analgesia including abnormal coagulation status, local infection, pre-existing neurological deficits of the torso or lower limbs, and spinal disease, (5) allergy to study medication, (6) pregnancy, (7) cognitive impairment, and (8) insufficient comprehension of the Dutch QoR-15 questionnaire.

### Who will take informed consent? {26a}

Informed consent will be obtained by an anesthesiologist during the preoperative anesthesiology consultation. An informed consent form will be signed by both the patient and the anesthesiologist.

### Additional consent provisions for collection and use of participant data and biological specimens {26b}

The informed consent form includes consent for collection, analysis, and anonymized reporting of participant data. No additional consent provisions are required as no biological specimens are collected.

## Interventions

### Explanation for the choice of comparators {6b}

The control group will receive thoracic epidural analgesia (TEA). This is considered the gold standard analgesic technique for video-assisted thoracoscopic surgery (VATS) [1].

### Intervention description {11a}

#### Investigational treatment

During the study, patients will receive standard preoperative care. Both techniques will be performed after placement of an intravenous (IV) line and application of standard vital sign monitors (non-invasive blood pressure, electrocardiogram, and oxygen saturation). The TEA and the ESP block will be performed before the start of surgery, according to institutional protocol. The ESP block will be performed under ultrasound guidance and the thoracic epidural will be performed using the conventional landmark-guided technique in line with currently accepted practice. All interventions will be performed by consultant anesthesiologists experienced in the technique.

##### Intervention group: continuous ESP block

The ESP block will be performed as described by Forero et al. [6]. The patient will be placed in the lateral or sitting position. An ultrasound probe will be placed in a longitudinal position 2–3 cm lateral of the vertebral column. The erector spinae muscles will be identified in relation to the ipsilateral fifth thoracic vertebra (T5) transverse process. A Tuohy needle will be inserted with an in-plane technique in a caudal to cephalad direction until bony contact with the transverse process is obtained. Hydrodissection with normal saline will be performed to identify and open up the correct plane for injection. A loading dose of ropivacaine will be injected followed by the insertion of an 18-g catheter 5 cm beyond the needle tip. Patients over 70 kg will receive 200 mg ropivacaine (40 ml), patients 50–70 kg will receive 150 mg ropivacaine (40 ml), and patients under 50 kg will receive ropivacaine 3 mg/kg (40 ml). No further local anesthetic will be administered intra-operatively. Following the end of surgery, a continuous infusion of 5 ml/h of bupivacaine 0.125%, supplemented by a 10-ml bolus injection every 3 h, will be administered through the ESP catheter

##### Control group: continuous thoracic epidural analgesia

The epidural catheter will be inserted preoperatively at the T5-T7 vertebral level; the exact level will be at the discretion of the attending anesthesiologist. A loading dose of bupivacaine 0.25% (max 10 ml) or ropivacaine 0.75% (max 10 ml) will be administered, followed by an intraoperative infusion of bupivacaine 0.125 % with sufentanil 1 μg/ml or ropivacaine 0.2% with sufentanil 0.5 μg/ml. The choice of ropivacaine or bupivacaine will be at the discretion of the attending anesthesiologist, in line with their individual institution’s established protocol for thoracic epidural infusion. Both ropivacaine 0.2% and bupivacaine 0.125% have been shown to be equipotent in multiple studies [18].

Postoperatively, we will not measure blocked sensory dermatomes in both ESP and TEA group as part of standard treatment. For TEA, it is our current practice to measure blocked sensory dermatomes only in patients who have a NRS > 4. For ESP block patients, other studies showed highly variable sensory blockade [19, 20]. As we see no added benefit, we decided not to determine the number of blocked dermatomes.

#### General treatment regimen

Induction of anesthesia, intraoperative hemodynamic management, and mechanical ventilation will follow current standards of care for both groups. In the operating room, general anesthesia is induced with propofol, sufentanil for analgesia, and rocuronium for paralysis. The trachea is intubated, and the lungs are mechanically ventilated with pressure-regulated volume-controlled ventilation. After induction, general anesthesia is maintained with a propofol infusion and supplemented as needed by additional boluses of sufentanil for intraoperative analgesia. A radial arterial line and an internal jugular central venous line will be inserted at the discretion of the attending anesthesiologist. A urinary catheter is inserted for the patients in the TEA group. Patients in the ESP group will receive a urinary catheter only if indicated; for example, by the expected hemodynamic instability or length of the surgery. If patients in the ESP group receive a urinary catheter, it will be removed at the end of surgery. All patients without urinary catheter will receive a bladder scan in the post-anesthetic care unit (PACU) before discharge to the ward as part of standard postoperative care. Cefuroxime 1500 mg is administered prior to incision.

#### Postoperative analgesia regimen

##### Intervention group: erector spinae plane

Patients in the intervention group will receive continuous ESP analgesia with an infusion of 5 ml/h bupivacaine 0.125% and a nurse administered bolus of 10 ml bupivacaine 0.125% every 3 h. The nurse will get an automated order to give the bolus at regular 3-h intervals. The patients will also receive a patient-controlled intravenous analgesia (PCIA) pump with piritramide and droperidol. Patients access to systemic opioids on an as-needed basis in the intervention group is included as part of the multimodal analgesia regimen recognizing the fact that patients in the control group are also receiving opioids in the epidural infusion. Settings of the PCIA pump will be according to local institutional protocols. When the ESP regimen does not provide a NRS < 4, we will adjust the parameters of the PCA to deliver higher doses.

##### Control group: thoracic epidural analgesia

Patients in the control group will receive TEA through a continuous epidural analgesia (CEA) pump with either bupivacaine 0.125% + sufentanil 1 μg/ml (8–12 ml/h with the possibility of a bolus of 4 ml every hour) or ropivacaine 0.2% + sufentanil 0.5 μg/ml (rate of 5 ml/h with a 2 ml bolus on demand with a lock-out of 20 min) according to local institutional protocols. When CEA does not provide a NRS < 4, an extra bolus of 5 ml of bupivacaine 0.125% + sufentanil 1 μg/ml or ropivacaine 0.2% + sufentanil 0.5 μg/ml will be given.

Discontinuation of ESP or CEA is planned on the morning of day 2, after APS assessment, unless side effects or safety issues mandate discontinuation or removal earlier. Patients will receive a daily visit by the acute pain service (APS) team, who will convert the patients to oral analgesics (paracetamol, NSAID and oxycodone) after removal of ESP or CEA.

#### Use of co-interventions

All patients will receive the standard postoperative multimodel non-opioid pain treatment with paracetamol and NSAIDs following institutional protocol. In the PACU, intravenous piritramide will be titrated until NRS ≤ 4. If this is insufficient, intravenous clonidine 1 mcg/kg will be administered.

The APS team will visit the patient daily and adjust the analgesic regimen as described above to manage inadequate analgesia. They will systematically screen for side effects, failure of analgesic technique, need for rescue medication, and need for specialist intervention.

#### Summary of known and potential risks

In a prospective audit report from the UK, the incidence of permanent injury from neuraxial anesthesia was 4.2 per 100,000 [21]. Complications such as total spinal, subdural injection, nerve injury, spinal–epidural hematoma, and infection are described [22]. The incidence of spinal–epidural hematoma was 1:18,000 in a retrospective study in 2004 [23].

For the erector spinae plane block, there is significantly less literature on complications. The most important risk of this block is that of local anesthetic systemic toxicity (LAST), as large volumes of local anesthetic agents are injected. In a case series published by Tulgar et al. of 182 patients undergoing ESP block, complications were reported by 0.22% (*n* = 4) patients [24]. They were all minor reactions, for example hypotension, and specifically no cardiac events [25].

In our current expertise, which follows the available literature, we perceive that ESP block is safe compared to the standard treatment, epidural analgesia. It is conducted in an environment with extensive monitoring of vital parameters and direct presence of experts in the field able to provide immediate support if required. The dosing with catheters in the postoperative phase resembles treatment, which is current standard practice in peripheral nerve blocks and postoperative epidural analgesia.

### Criteria for discontinuing or modifying allocated interventions {11b}

We will exclude patients to participate in the study in case of failure of primary insertion of the ESP or TEA catheter. This will be documented as technical failure (Table 1). Secondary failures, such as catheter dislodgement, migration, or leakage leading to premature removal of the catheter will be documented. Need for rescue medication and need for specialist intervention will also be reported. When the surgical technique changes intraoperatively, e.g., conversion to open procedure, the patient will be replaced and will not be used in the intention to treat analysis.

### Strategies to improve adherence to interventions {11c}

The APS team will visit the patient daily and ensure that the prescribed analgesic regimen is being adhered to. They will adjust the analgesic regimen as described above to manage inadequate analgesia. They will systematically screen for side effects, failure of analgesic technique, need for rescue medication, and need for specialist intervention.

### Relevant concomitant care permitted or prohibited during the trial {11d}

The postoperative pain management strategy is outlined in the study protocol. Concomitant care of any other kind is permitted during the trial, if necessary, and there are no specific prohibitions.

### Provisions for post-trial care {30}

The sponsor/investigator has liability insurance, which is in accordance with article 7 of the WMO. This insurance provides cover for damage to research subjects through injury or death caused by the study.

### Outcomes {12}

#### Primary endpoint

The primary outcome in this study is the Quality of Recovery-15 (QoR-15) score. This score is a compound measure of overall postoperative patient recovery. The score comprises a 15-item questionnaire that provides an evaluation across five dimensions: patient support, comfort, emotions, physical independence, and pain. The questionnaire has been validated extensively and a minimal clinically important difference has been defined [26]. We will measure the QoR-15 score before surgery as a baseline and on postoperative days (POD) 1 and 2 as these questions relate to the 24-h period covering the day of the surgery and the first postoperative day respectively.

#### Secondary endpoints

Secondary endpoints are Numerical Rating Scales (NRS) score preoperatively and on POD 0, 1, and 2 (assessed at rest and when moving (coughing) in the morning and in the evening), length of hospital stay (LOS), failure of analgesic technique (defined as catheter failure, need for specialist intervention and/or need for rescue medication, see Table 1), the postoperative morphine-equivalent consumption on POD 0, 1, and 2, itching, nausea and vomiting, the total operative time (recorded as total time spent in the operating room, anesthetic time, surgical time), complications related to surgery (e.g., bleeding, surgical site infection, conversion to open procedure), perioperative hypotension despite fluid boluses or vasopressor bolus (defined by use of continuous infusion of vasopressors) complications related to the study intervention (e.g., epidural hematoma and abscess or local anesthetic toxicity), duration of bladder catheterization, and time of first assisted mobilization (> 20 m and to sitting in a chair).

#### Other study parameters

Upon inclusion, we will collect gender, age, weight, height, American Society of Anesthesiologists (ASA) risk score, type of surgery, and opioids in home medication.

### Participant timeline {13}

**Fig. 1:**
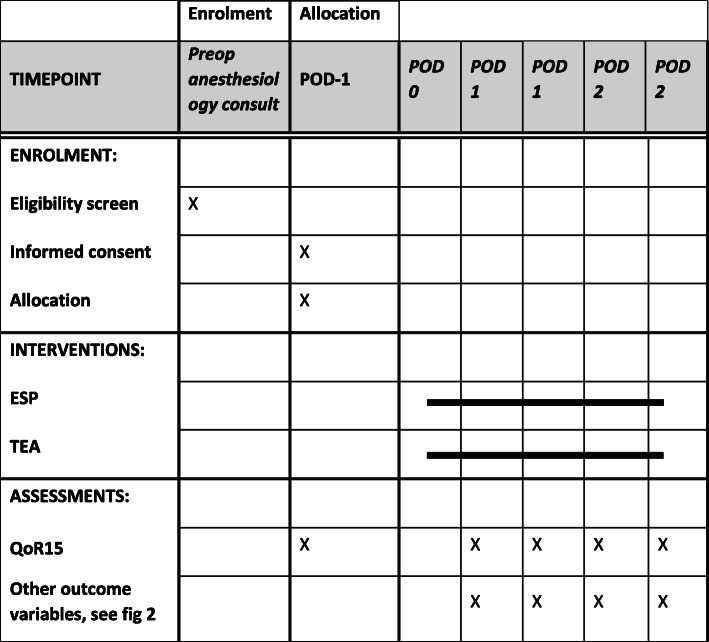
Schedule of enrolment, interventions, and assessments

**Fig. 2:**
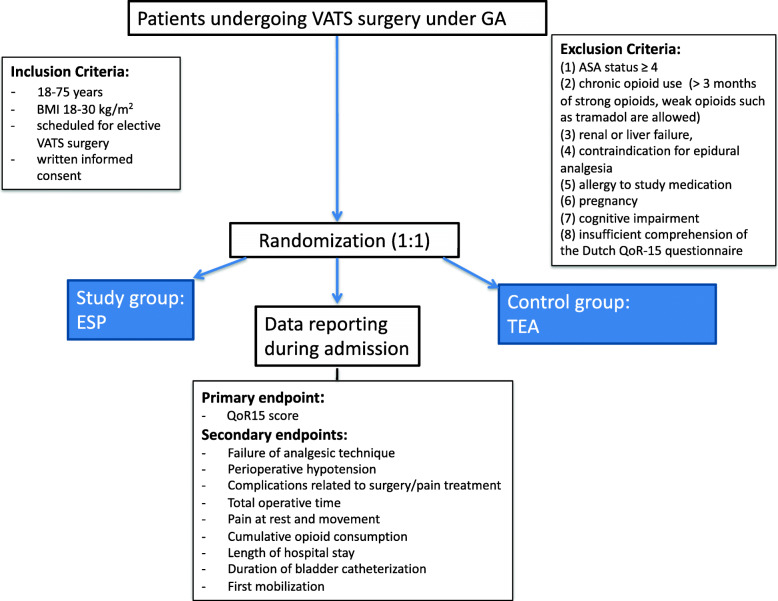
Flow chart of the study design. GA, general anesthesia; VATS, video-assisted thoracoscopic surgery; BMI, body mass index; ASA, American Society of Anesthesiologists; ESP, continuous erector spinae plane analgesia; TEA, thoracic epidural analgesia; QoR15, Quality of Recovery 15 Score

Patients will be screened and given oral and written information about the study during the preoperative anesthesiology consultation. They are asked to participate in the study on the day before surgery. They will be randomized into an ESP group and a TEA group. A baseline assessment of the QoR-15 upon inclusion will be done. Patients will undergo placement of TEA of ESP catheter just prior to surgery. After surgery, on the recovery ward or ICU, the NRS score will be taken by the recovery nurse or ICU nurse. Pain medication will be given, according to this protocol. Patients will receive a daily visit by the acute pain service (APS) team until they can be converted to oral analgesics: paracetamol, NSAID, and oxycodon. Patients will be followed until POD2 or until (earlier) discharge from the hospital. A flowchart of the study design is presented in Figs. 1 and 2.

**Table 1:** Definitions of technical complications

Technical failure	Failure to place a catheter
Catheter failure	Catheter dislodgement, migration, or leakage, leading to premature removal of the catheter.
Need for rescue medication	Patients remain painful (NRS > 4) after maximum adjustments possible
Need for specialist intervention	Suboptimal functioning of CEA/ESP on ward leading to supervision by an anesthesiologist and/or minor adjustments

### Sample size {14}

Based on the study published by Myles, we assumed the standard deviation of the QoR15 to be 18 for video-assisted thoracic surgery (intermediate extent of surgery) [26]. From the same study, a non-inferiority limit of 13 was assumed as Myles et al. determined that this value represents “minimal change” for the QoR15. Using a one-sided alpha of 0.05, a standard deviation of 18, and a power of 95%, 42 patients are necessary in each group. With a dropout rate of 7%, the total number of patients to randomize is 90. The two hospitals have a joint recruitment pool of 180 VATS per year and expect 60% of the patients to be both eligible and willing to participate. We aim to complete inclusion within a ± 12–18-month period. A total of 90 patients are being randomly allocated to ESP (study group) or TEA (control group).

### Recruitment {15}

In both study centers, adult patients scheduled for elective VATS for lobectomy or wedge resection under general anesthesia will be screened for eligibility at the preoperative anesthesiology consultation. If they are eligible to participate in the study, they will receive oral and written information. The day before surgery, the patients will be asked to participate in the study by an anesthesiologist. When they decide to participate, informed consent forms will be signed by both the patient and anesthesiologist. Enrollment will continue until the total number of 90 patients is reached. We estimate the recruitment period to take 12–18 months.

### Assignment of interventions: allocation

#### Sequence generation {16a}

In this open-label study, patients will be randomly allocated in a 1:1 ratio to receive either an ESP block (study group) or a TEA block (control group) by using a predetermined computer-generated randomized schedule. Only the investigators will have access to this list. Permutated block randomization with varying permuted block sizes will be used to divide the lobectomy and wedge resections in homogenous strata to manage for any surgical variation. Separate randomization lists will be used for the two hospitals, to reduce bias. This blocked randomization list will be created with Sealed Envelope™ software.

#### Concealment mechanism {16b}

Participants will be randomized using Research Manager, My Data Manager which is an online, central randomization service within the program Research Manager. Allocation concealment will be ensured, as the service will not release the randomization code until the patient has been recruited into the trial, which takes place after all baseline measurements have been completed.

#### Implementation {16c}

The person who will generate the allocation sequence is not involved in the study. The attending anesthesiologist in the preoperative anesthesiology clinic will screen and enroll participants. The researchers will assign participants to interventions, according to the randomization.

### Assignment of interventions: blinding

#### Who will be blinded {17a}

Blinding of the trial participants and study personnel engaged in patient care to group allocation is not possible due to the different clinical characteristics and invasive nature of the two study interventions. Statistical analysis will be performed by a third-party who is not otherwise involved in the conduct of the study; they will be fully blinded to the hypothesis of the study and to the group allocation.

#### Procedure for unblinding if needed {17b}

The design is open label so a procedure for unblinding is not required.

### Data collection and management

#### Plans for assessment and collection of outcomes {18a}

Patients’ demographical data will be collected upon inclusion assessment during the preoperative anesthesiology consultation. The attending anesthesiologist will collect data with regard to the anesthesia and surgical procedure. Nurses will collect the data at the Post Anesthesia Care Unit (PACU). The APS team will collect QoR15 scores, opioid consumption, NRS scores, and adverse events. The amount and frequency of the opioid usage will be extracted out of the PCA pump. NRS scores and additional administration of analgesia will be extracted out of the medical chart of the patient as collected by the nurses at the ward. Complications will be assessed on POD 2 or earlier at discharge. Some of the data will be registered on paper. After termination of the trial, the data will be directly registered in the software program My Research Manager.

#### Plans to promote participant retention and complete follow-up {18b}

To reduce non-retention, the APS team will encourage the participants to complete the questionnaires. Also, most data except for the questionnaires is registered on a regular basis for all patients who have epidural analgesia; therefore, this limits registration burden and prevents missing data.

Non-adherence to protocol is described in the “Criteria for discontinuing or modifying allocated interventions {11b}” section. All non-adherence will be documented and reported.

#### Data management {19}

The monitoring team of the Catharina Hospital Eindhoven will provide the monitoring and quality assurance of this study. Data entry, coding, and storage will be done according to the GCP standards. Publication of data will be done anonymously.

### Confidentiality {27}

The data of each patient will be noted on an individual case report form. Data will be coded using a numerical code, the key to this code is only available to the research team and is stored in the investigator site file in accordance with the Dutch law “Algemene Vordering Gegevensbescherming (AVG; Personal Data Protection Act)” and GCP. All patient data will be handled confidentially and anonymously. Data will then be inserted into a database (GCP validated), and a second investigator will control correctness of entries. All data, including case report forms and consent forms, will be stored for 15 years after completion of the study. Data, both anonymous or not, will always be stored securely, in a locked cabinet (hard copy) or on password secured computers.

### Plans for collection, laboratory evaluation, and storage of biological specimens for genetic or molecular analysis in this trial/future use {33}

N/A, biological specimens are not used in this study.

## Statistical methods

### Statistical methods for primary and secondary outcomes {20a}

All statistical analyses will be done in consultation with a senior statistician. The primary analysis will be an intention to treat analysis. Secondary analyses will include a per protocol analysis to identify any protocol deviations or other safety signals. Continuous data will be presented as mean and standard deviation or median and interquartile range. Normality of distributions for continuous variables will be assessed with skewness and kurtosis measures. Continuous data will be assessed using a Student’s *t* test if they are normally distributed or a Mann-Whitney *U* test if otherwise. Dichotomous data will be presented in percentages. Categorical data will be analyzed using a chi-square test or Fisher exact test.

#### Primary study parameter(s)

We will compare the (mean) score of QoR15 of each day separately between the standard and the intervention group using the Student’s *t* test. Repeated measures analysis will be used to assess changes over time in the study parameters.

#### Secondary study parameter(s)

Continuous data (postoperative morphine-equivalent consumption per day, postoperative NRS score on POD 1 and 2; length of hospital stay, requirement of rescue medication, failure of anesthetic technique, total operative time, complications related to surgery or pain treatment, duration of bladder catheterization, first assisted mobilization to chair and > 20 m, 30-day postsurgical evaluation) will be assessed using a Student’s *t* test if they are normally distributed or a Mann-Whitney *U* test if otherwise. Categorical data (e.g., gender) will be analyzed using a chi-square test or Fisher exact test. The mean difference between the two groups will be presented together with the 95% confidence interval.

### Interim analyses {21b}

An interim-analysis will be performed after randomization of 50% of the patients. This analysis will be performed by the Education and Research team of our hospital, more specifically a professional in statistical analysis, who is not engaged in the anesthesiology practice or with our (study) patients. Criteria for terminating the study prematurely are indicators of insufficient patient comfort and are defined as mean NRS > 6 of all patients during more than 2 consecutive measurements in the study group in the first 24 h or mean QoR15 < 83 in the study group on POD2.

### Methods for additional analyses (e.g., subgroup analyses) {20b}

There will be no subgroup or adjusted analysis.

### Methods in analysis to handle protocol non-adherence and any statistical methods to handle missing data {20c}

Primary analysis will be an intention to treat analysis. Secondary analysis will include a per protocol analysis. Within the 7% drop out safety buffer, there will be no replacement of study patients. A drop out is defined as a patient not completing the full duration of the study. When the surgical technique changes intra operatively, e.g., conversion to open procedure, the patient will be replaced and will not be used in the intention to treat analysis.

There will be no follow-up of patients withdrawn from the treatment if they withdraw before at least the QoR15 score on POD1 is registered. In that case, the patient will be replaced after we exceeded the 7% withdrawal safety buffer. Data of the dropouts with a QoR15 score on POD1 will be used in the intention to treat analysis.

### Plans to give access to the full protocol, participant level-data, and statistical code {31c}

The datasets generated during and/or analyzed during the current study will be made available from the corresponding author on reasonable request and will only be accessible to personnel involved in the trial.

## Oversight and monitoring

### Composition of the coordinating center and trial steering committee {5d}

The Catharina Hospital is the coordinating center. Arthur Bouwman is the lead investigator. Renee van den Broek is the main researcher at the Catharina Hospital. Seppe Koopman is the main researcher at the Maasstad Hospital. They are responsible for identification, recruitment, data collection, and completion of CRFs, along with follow-up of study patients and adherence to study protocol. A senior statistician will be consulted for statistical analysis. All protocol contributors will draft and/or revise the manuscript.

### Composition of the data monitoring committee, its role and reporting structure {21a}

The Monitoring team of the Catharina Hospital Eindhoven will provide the monitoring and quality assurance of this study. The monitor for this study is Sylvie Kolfschoten, department of research and education, Catharina Hospital, the Netherlands.

This study has a low risk, and there is no need for a DSMB (Data Safety Monitoring Board). The technique used does not pose extra safety risks compared to the standard treatment and is conducted in an environment with extensive monitoring of vital parameters and direct presence of experts in the field able to provide immediate support if required. The dosing with catheters in the postoperative phase resembles treatment, which is current standard practice in peripheral nerve blocks and postoperative epidural analgesia.

### Adverse event reporting and harms {22}

In accordance to section 10, subsection 4, of the WMO, the sponsor (in this investigator-initiated study, the investigator) will suspend the study if there is sufficient ground that continuation of the study will jeopardize subject health or safety. The investigator will notify the accredited MREC (Medical Research Ethics Committee) without undue delay of a temporary halt including the reason for such an action. The study will be suspended pending a further positive decision by the accredited MREC. The investigator will take care that all subjects are kept informed.

Adverse events are defined as any undesirable experience occurring to a subject during the study, whether or not considered related to the trial procedure. All adverse events reported spontaneously by the subject or observed by the investigator or his staff will be recorded.

A serious adverse event is any untoward medical occurrence or effect that:

- Results in death;
- Is life threatening (at the time of the event);
- Requires hospitalization or prolongation of existing inpatients’ hospitalization;
- Results in persistent or significant disability or incapacity;
- Is a congenital anomaly or birth defect; or
- Any other important medical event that did not result in any of the outcomes listed above due to medical or surgical intervention but could have been based upon appropriate judgment by the investigator.

An elective hospital admission will not be considered as a serious adverse event.

The investigator will report all SAEs to the sponsor without undue delay after obtaining knowledge of the events, except for the following SAEs: surgical or other complications unrelated to the anesthetic procedure.

The sponsor will report the SAEs through the web portal ToetsingOnline to the accredited MREC that approved the protocol, within 7 days of first knowledge for SAEs that result in death or are life threatening followed by a period of maximum of 8 days to complete the initial preliminary report. All other SAEs will be reported within a period of maximum 15 days after the sponsor has first knowledge of the serious adverse events.

#### Follow-up of adverse events

All AEs will be followed until they have abated or until a stable situation has been reached. Depending on the event, follow-up may require additional tests or medical procedures as indicated and/or referral to the general physician or a medical specialist. SAEs need to be reported till end of study within the Netherlands, as defined in the protocol

### Frequency and plans for auditing trial conduct {23}

The Monitoring team of the Catharina Hospital Eindhoven will provide the monitoring and quality assurance of this study. The monitor will perform an initiation visit and will audit the overall quality and completeness of the data, examine source documents, and confirm that the clinical centers have complied with the requirements of the protocol. The monitor will verify that all adverse events were documented in the correct format and are consistent with protocol definition.

### Plans for communicating important protocol amendments to relevant parties (e.g., participants, ethical committees) {25}

Amendments are changes made to the research protocol after a favorable opinion by the accredited ethics committee. All amendments will be notified to the ethics committee.

### Dissemination plans {31a}

The trial’s results will be submitted to a peer-reviewed journal regardless of the outcome.

## Discussion

Although the ESP block is gaining popularity as analgesia in thoracic surgery, there has been limited study of its clinical efficacy compared with the current gold standard of thoracic epidural analgesia (TEA). However, these preliminary results are promising [10, 11, 13, 27–29]. This prompted us to speculate that ESP block incorporated into a multimodal analgesia regimen may have a similar analgesic profile to TEA and in turn to conduct a trial to compare these two techniques and further validate the theory from a clinical aspect.

The expected results will provide clinical evidence to verify the analgesic mechanism of ESP and promote its application in minimally invasive thoracic surgery. Furthermore, if the efficacy of ESP in a multimodal analgesia regimen is equal to that of TEA, ESP could replace TEA in minimally invasive thoracic surgery because it is relatively safe and convenient to perform, while TEA carries risks of failure, dural puncture, and epidural abscess or hematoma [14, 21, 22, 30]. Furthermore, unlike TEA, ESP does not require routine urinary catheterization to manage urinary retention and thus facilitates the early mobilization of the patient after surgery.

The proposed trial is a prospective, randomized trial with rigorous methodology to avoid potential risk of bias. Since the nature of the intervention makes it impossible for the participant, the investigator performing the block, and the outcome assessor to be blinded, only the statistician will be blinded to the allocation to keep the data analysis unbiased.

In contrast to epidural analgesia, ESP analgesia can only induce a unilateral sensory block of thoracic dermatomes. Nonetheless, this unilateral thoracic block is expected to achieve adequate control of chest pain after minimally invasive thoracic surgery, especially when combined with patient-controlled intravenous opioid analgesia as an escape for breakthrough discomfort [11–13]. If ESP analgesia in fact achieves adequate pain control, the avoidance of epidural-related side-effects and absence of urinary catheterization likely facilitates rapid recovery after minimally invasive thoracic surgery, which is the primary interest of this study. Since postoperative recovery is multifactorial, a composite endpoint was considered to be most appropriate and therefore the QoR-15 questionnaire was chosen as primary outcome. The QoR-15 consists of 15 questions that are divided in separate sections that aim to evaluate the presence and extent of pain, symptoms, comfort, emotional well-being, physical independence, and satisfaction with treatment [31]. As the hypothesized advantages of ESP analgesia are expected to impact several of these sections, the composite QoR-15 score is considered to be a good parameter for postoperative recovery.

Our evaluation through the QoR15 score matches the call for more patient-centered outcomes in ESP block studies [15, 32]. The reduction in pain scores itself may not equate to an improvement in patients experience. Factors other than only analgesia, such as general well-being, nausea, itching, being able to get out of bed, appetite, and the need for a bladder catheter also matter in the patients’ perspective on good recovery and contribute to enhanced recovery and earlier hospital discharge.

If our trial yields positive results, there is potential that ESP could be recommended as an alternative block for postoperative analgesia in minimally invasive thoracic surgery, to circumvent the need for TEA.

## Trial status

This document is based on version 5 of the original protocol, approved by the MET-U (ethics committee) on 26 February 2020, registration number R18.022. We anticipate randomizing the first patient in July 2020 and plan to complete the study in December 2021.

## Data Availability

The lead investigator AB and both RB and BV will have access to the final trial dataset. Other contributors of this protocol are allowed access to the final trial dataset upon reasonable request.

## Abbreviations

ESP: Erector spinae plane
TEA: Thoracic epidural analgesia
VATS: Video-assisted thoracoscopic surgery
QoR15: Quality of recovery
NRS: Numeric Rating Scale
LOS: Length of stay
POD: Postoperative day
ASA: American Society of Anesthesiology
NSAID: Non-steroidal anti-inflammatory drug
INR: International normalized ratio
NIBP: Non-invasive blood pressure
ECG: Electrocardiogram
BMI: Body mass index
CEA: Continuous epidural analgesia
APS: Acute pain service
LAST: Local anesthetic systemic toxicity
PACU: Post anesthesia care unit
PCIA: Patient controlled intravenous analgesia
AE: Adverse event
SAE: Serious adverse event
GCP: Good clinical practice
DSMB: Data Safety Monitoring Board
MREC: Medical Research Ethics Committee
